# Graft protective effect and induction of CD4^+^Foxp3^+^ cell by Thrombomodulin on allograft arteriosclerosis in mice

**DOI:** 10.1186/s13019-018-0731-8

**Published:** 2018-05-21

**Authors:** Enzhi Yin, Shigefumi Matsuyama, Masateru Uchiyama, Kento Kawai, Masanori Niimi

**Affiliations:** 1 Department of Surgery, Teikyo University, 2-11-1 Kaga, Itabashi-ku, Tokyo, 173-8605 Japan; 2 Department of Cardiovascular Surgery, The 2nd Affiliated Hospital of Harbin Medical University, Harbin, China; 3 Department of Cardiovascular Surgery, New Tokyo Hospital, Chiba, Japan; 4 Department of Cardiovascular Surgery, Teikyo University, Tokyo, Japan; 5 Transplantation Research Immunology Group, Nuffield Department of Surgical Sciences, University of Oxford, John Radcliffe Hospital, Oxford, UK

**Keywords:** Thrombomodulin (CD141), graft protection, CD4^+^Foxp3^+^ cell, heart transplantation

## Abstract

**Background:**

Thrombomodulin (TM) is a promising therapeutic natural anti-coagulant, which exerts the effects to control disseminated intravascular coagulation. However, little is known whether TM on micro-vessels could play an important role in the regulation of intimal hyperplasia. We investigated the vessel-protective effect of TM in the survival of fully major histocompatibility complex (MHC)-mismatched murine cardiac allograft transplantation.

**Methods:**

CBA recipients transplanted with a C57BL/6 heart received intraperitoneal administration of normal saline or 0.2, 2.0, and 20.0 μg/day of TM for 7 days (*n* = 5, 7, 11, and 11, respectively). Immunohistochemical and fluorescent staining studies were performed to determine whether CD4^+^Foxp3^+^ regulatory T cell were generated at 2 and 4 weeks after grafting. Morphometric analysis for neointimal formation in the coronary arteries of the transplanted allograft was conducted at 2 and 4 weeks after grafting.

**Results:**

Untreated CBA recipients rejected C57BL/6 cardiac grafts acutely (median survival time [MST], 7 days). CBA recipients exposed with the above doses had significantly prolonged allograft survival (MSTs, 17, 24 and 50 days, respectively). Morphometric assessment showed that intimal hyperplasia was clearly suppressed in the left and right coronary arteries or allografts from TM-exposed recipients 2 and 4 weeks. Immunohistochemical studies at 2 weeks showed more CD4^+^Foxp3^+^ cells and lower myocardial damage in the allografts from TM-exposed recipients. Notably, fluorescent staining studies demonstrated that TM-exposed recipients 4 weeks post-engraftment had strong aggregation of CD4^+^Foxp3^+^ cells in the intima of the coronary arteries of the cardiac allografts.

**Conclusions:**

TM may prolong the survival of fully MHC-mismatched cardiac allografts through suppressing intimal hyperplasia and inducing the accumulation of regulatory CD4^+^Foxp3^+^ cells within coronary arteries.

## Background

The coagulation cascade is a multi-step process that can cause efficient clotting even with minimal coagulatory activation. Hemostatic factors such as platelets and coagulation factors are produced in surplus of as much as ten times more than required for effective clotting; thus, even if the number of platelets were to decrease to one tenth, bleeding rarely occurs. Conversely, anti-coagulation mechanisms are much less dynamic due to the lack of a similar enhancement system and minimal circulating anti-coagulants in comparison. In comparison, even diminishing the anticoagulants to half its normal amount will render it ineffective in preventing the formation of clots. Therefore, exogenous anti-coagulants (i.e. heparin sulfate, tissue plasminogen activator, prostacyclin, nitric oxide, and thrombomodulin) are frequently administered to prevent excessive coagulation. In particular, thrombomodulin (TM) has recently been used as a promising therapeutic natural anti-coagulant drug within clinical trials.

Discovered by Esmon et al. in 1982, TM (CD141) is expressed on the endothelial cell surface of all vessels [1]. The anti-coagulatory effects of TM can alleviate disseminated intravascular coagulation (DIC) induced by hematologic malignancy and severe infections such as sepsis [2]. It was demonstrated that TM forms a complex with thrombin that activates protein C (PC), which then inactivates Va and VIIIa factor to regulate subsequent thrombin formation [1]. After the anti-coagulatory mechanism of TM was gradually elucidated, use of recombinant human soluble TM (rTM) for the treatment of DIC and severe infections was approved in Japan in 2008, leading to many mega-studies on TM. For instance in Japan in 2007, a randomized, double-blind clinical, and phase III trial on the effects of rTM and low-dose heparin on DIC associated with hematologic malignancy or infection demonstrated that rTM could improve DIC resolution rate and clinical course of bleeding symptoms [2]. In Belgium in 2013, another randomized, double-blind, phase IIa trial on the effects of rTM on DIC associated with sepsis showed that D-dimer and prothrombin fragments, which are markers of the fibrinolytic system, were lower in the rTM group than in the placebo group [3]. Motivated by favorable outcomes in clinical trials and the promising effectiveness of TM on the control of coagulation and inflammation under severe conditions, we hypothesized that TM-based therapy may be effective in mediating acute rejection of heart transplantation by regulating or suppressing conditions such as myocardial destruction and micro-vasculopathy.

Generally, TM is thought to be expressed on the endothelial cell surface of all vessels. However, little is known whether TM expression on micro-vessels could play a role in the regulation of microcirculation disturbance or allograft vessel protection. In a study on pediatric heart transplant recipients, serum levels of post-transplant TM were elevated after transplantation, except in patients with severe chronic allograft vasculopathy who displayed significantly lower levels [4]. Moreover, some reports indicated the effects of TM to transplantation-associated microangiopathy after hematopoietic stem cell transplantation [5]. Taken together, these insights may indicate that the manipulation of TM concentration can mediate progression of angiopathy and that fluctuation of natural TM can be used as a clinical biomarker after transplant. Therefore, in this study we investigated whether coagulopathy and microvasculopathy play a role in the pathogenesis of allograft rejection and also whether TM-based treatment had vessel-protective effects in a model of fully MHC-mismatched murine cardiac allograft transplantation.

## Methods

### Animals

Male CBA (H2^k^) and C57BL/6 (H2^b^, B6) mice that were 8 to 12 weeks of age were purchased from Sankyo Ltd. (Tokyo, Japan), housed in conventional facilities at the Biomedical Services Unit of Teikyo University, and used in accordance with the guidelines for animal experimentation approved by Medicine Animal Ethics Committee of Teikyo University (13-023, 7/11/2013) and the “Principles of laboratory animal care” (NIH publication, vol. 25, no. 28, revised 1996).

### Heart transplantation

**Fig. 1:**
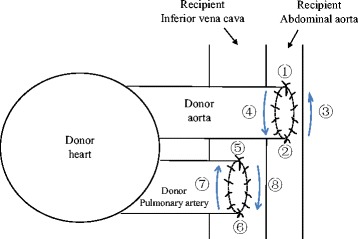
Drawing of anastomoses procedure in the heterotopic cardiac grafts

Heart transplantation was conducted as described previously [6]. Briefly, all heart transplantation in mice were performed by one operator who can achieve a 98% successful rate. In this experiment (donor: male B6; recipient: male CBA), fully allogeneic cardiac transplant model with transplanted heart beating over 3 days was considered as a successful and recordable model. The operation began with preparation of the recipient animal. Under an operation microscope at 15× magnification the inferior vena cava and abdominal aorta below the renal vessels were dissected free, as ligating lumbar veins and arteries between sufficient recipient vessels for anastomosis. Two Scoville-lewis clamps (Downs Surgical Ltd., London, UK) were placed around both aorta and vena cava in preparation for later occlusion of the vessels. In preparation of the donor animal, inferior vena cava, azygos vein, and the superior vena cava were ligated with 7-0 silk sutures in order. The aorta and pulmonary artery were separated and divided as far distally as possible. The pulmonary veins were ligated as a group with a single 7-0 silk sutures. The donor aorta was sutured end-to-side to the recipient abdominal aorta using 10-0 surgical suture (Kyowa Precision Instruments Crop., Tokyo, Japan) (Fig. 1). Subsequently the pulmonary artery was anastomosed to the recipient inferior vena cava. Upon release of the clamps the heart begun to fibrillate and usually within a few minutes it reverted to a sinus rhythm. Postoperatively, cardiac graft function was assessed daily by palpating the heart for evidence of contraction. Rejection was defined as complete cessation of the heartbeat and confirmed by direct visualization and histologic examination of the graft.

### Measurement of TM

Transplanted CBA recipients with beating B6 heart (untreated recipients) and naïve CBA mice were prepared to evaluate the concentration of serum TM in time series. The serums from untreated recipients on day 3, 5, 7, and 10 after grafting and naïve mice were obtained. Measurement of TM was assessed by using an ELISA for TM (R&D, #MTHBD0, Mouse Thrombomodulin/BDCA-3 Quantikine ELISA Kit, MN, USA) according to the manufacturer’s instructions.

### Exposure to TM

Transplanted CBA recipients were intraperitoneally exposed with one dose of 0.2, 2.0, and 20.0 μg/day of TM (Asahi Kasei Pharma Corporation, Tokyo, Japan) for the replenishment of TM from the day of cardiac transplantation to 7 days afterward (*n* = 7, 11, and 11, respectively). Each dose of TM was diluted with normal saline (1 ml). Transplant recipients in the control group were given intraperitoneal injections of normal saline (*n* = 5).

### Morphometric analysis of neointimal formation in allografts

The morphometric analysis was conducted as described previously [7]. Coronary artery cross-sections of donor B6 hearts from TM-exposed and untreated recipients 2 and 4 weeks after grafting were visualized by type IV collagen staining and analyzed by computer-assisted morphometry. The coronary arteries such as the left coronary artery (LCA) and the right coronary artery (RCA) were analyzed in the anatomical regions labeled clearly as the right ventricle and left ventricle as described in the previous paper [7]. All morphometric comparts including the lumen and intima were assessed by the image software (NanoZoomer Digital Pathology Virtual Slide Viewer, Hamamatsu Photonics, Hamamatsu, Shizuoka, Japan). The quantitative methodology was developed to assess the neointimal formation through the stenosis index (SI). SI was calculated as a percentage by dividing intimal area by the sum of lumen and intimal area.

### Histological, immunohistochemical (IHC) and fluorescent staining studies of harvest grafts

Cardiac allografts transplanted into untreated and TM-treated CBA recipients were removed 2 weeks after grafting and studied histologically and immunohistochemically. Frozen sections (4-μm thick) were cut, mounted on silane-coated slides, and stained with hematoxylin-eosin (HE). HE staining was assessed by grading with a semi-quantitative scale for the amount of mononuclear cell infiltration (0, no infiltration; 1, faint and limited infiltration; 2, moderate infiltration; 3, severe infiltration) [8, 9]. All graft heart slides were assessed blindly by unrelated three researchers.

IHC and fluorescent staining studies were performed to determine whether the myocardial function in the transplanted cardiac allografts was preserved and CD4^+^Foxp3^+^ regulatory T cells were generated. Results of double immunostaining of cardiac allografts were obtained 2 and 4 weeks after transplantation from mice exposed to 20.0 μg/day of TM. Additionally, the expression of TM (CD141) in cardiac allografts from TM-treated recipients was examined on 4 weeks after grafting. Fresh 4-μm-thick graft cryosections were fixed in ice-cold acetone and preincubated in Block Ace (Dainippon Pharmaceutical Co., Ltd., Tokyo, Japan). Samples were incubated with anti-CD4 monoclonal antibody (mAb) (RM4-5; BD Biosciences, San Jose, CA) and anti-CD141 (AF3894; R&D System) polyclonal antibody, or anti-Foxp3 mAb (FJK-16 s, eBioscience); incubated with alkaline phosphatase (ALP)-conjugated anti-rat Ig (712-055-153; Jackson ImmunoResearch Laboratories, West Grove, PA) for anti-CD4, with ALP-conjugated anti-goat Ig (705-055-003; Jackson ImmunoResearch Laboratories) for anti-CD141 and with ALP-conjugated anti-rabbit Ig (712-055-152; Jackson ImmunoResearch Laboratories) for anti-Foxp3; and developed blue with Vector Blue (Vector Laboratories, Burlingame, CA). Cryosections were then incubated with rabbit anti-mouse type IV collagen polyclonal antibody (LB1403; Cosmo Bio, Tokyo) and peroxidase-conjugated anti-rabbit Ig (55,693; Mitsubishi Chemical, Tokyo) and then developed brown with diaminobenzidine (Vector Laboratories). In IHC study, the number of infiltrating CD4^+^ and Foxp3^+^ cell in TM-treated recipient or untreated recipient was counted in the area of 400 μm × 400 μm. All graft heart slides were assessed blindly by unrelated researchers.

Triple fluorescent staining studies of cardiac allograft obtained 4 weeks after grafting from mice exposed to 20.0 μg/day of TM. Fresh 4-μm-thick graft cryosections were incubated with anti-Foxp3 mAb (FJK-16 s, eBioscience); then incubated with Alexa Fluor® 594-conjugated anti-rat Ig (A-11007, Thermo Fisher Scientific, Waltham, MA, USA) for anti-Foxp3 (shown as red in Fig. 7b). After blocking with normal rat Ig, cryosections were incubated with Alexa Fluor® 647-conjugated anti-CD4 mAb (RM4-5, BioLegend, San Diego, CA) (shown as green in Fig. 7b). Subsequently, cryosections were incubated with rabbit anti–mouse type IV collagen polyclonal antibody (LB1403; Cosmo Bio); then incubated with AMCA-conjugated anti-rabbit Ig (711-155-152, Jackson ImmunoResearch Laboratories) (shown as blue in Fig. 7b).

### Statistical analysis

Cardiac allograft survival in two experimental groups was compared by using log-rank test. In the immunohistochemical study, the difference between two groups was assessed by an unpaired Student’s t test.

A *P* value of less than 0.05 was regarded as significant.

## Results

### Fluctuation of TM

**Fig. 2:**
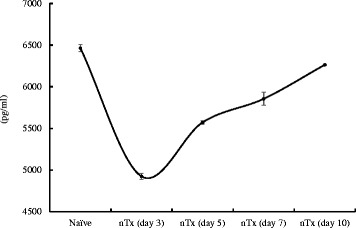
Measurement of Thrombomodulin (TM) in naïve CBA mice and untreated CBA recipients. The serums from untreated recipients on day 3, 5, 7, and 10 after grafting and naïve mice were obtained. nTx, untreated

TM concentration in the serum from untreated recipients on day 3, 5, 7, 10 and naïve mice was measured (*n* = 3). TM concentration decreased the most on day 3 after transplantation and gradually recovered afterward (Fig. 2). The level of TM concentration on day 10 was similar to that in naïve mice.

### Significant prolonged survival of cardiac allografts in mice exposed with TM

**Fig. 3:**
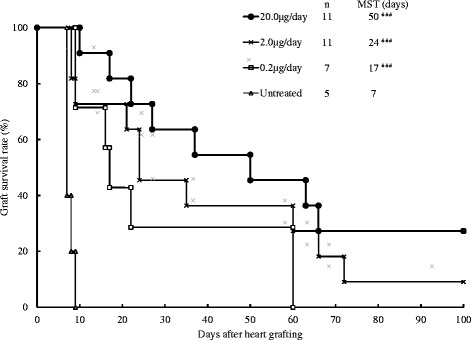
Cardiac graft survivals in CBA recipients of a C57BL/6 heart that were exposed to 0.2, 2.0, and 20.0 μg/day of Thrombomodulin (TM) from the day of transplantation to 7 days afterward. MST, median survival time. ###*p* < 0.001 compared with untreated group

Untreated CBA recipients rejected B6 cardiac grafts acutely (*n* = 5) (median survival time (MST), 7 days). CBA recipients exposed with 0.2 (*n* = 7), 2.0 (*n* = 11) and 20.0 μg/day (n = 11) of TM had significantly prolonged allograft survival in a dose-dependent manner (MSTs, 17, 24 and 50 days, respectively, *P* < 0.001 compared with untreated group; Fig. 3).

### Suppression of intimal hyperplasia in coronary arteries of donor B6 heart from TM-exposed CBA recipients

**Fig. 4:**
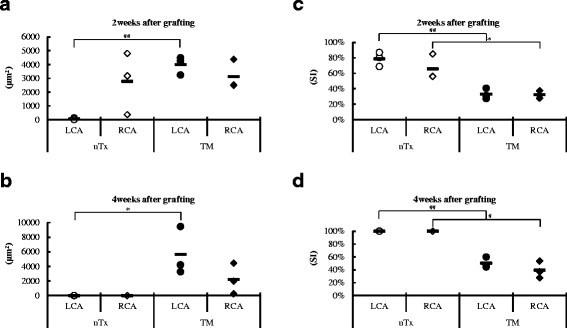
Morphometric analysis of neointimal formation in transplanted cardiac graft exposed with Thrombomodulin (TM) and normal saline. **a**, **b** Results of lumen area (μm^2^) in the left coronary arteries (LCA) and the right coronary arteries (RCA) of each group 2 and 4 weeks after grafting. **c**, **d** Results of SI (stenosis index) in LCA and RCA of each group 2 and 4 weeks after grafting. LCA, left coronary artery; nTx, untreated; RCA, right coronary artery; SI, stenosis index. *p < 0.05, #*p* < 0.01, and ##*p* < 0.005 for difference between two groups

Morphometric assessment showed the degree of neointimal formation and coronary stenosis in donor B6 hearts within CBA recipients exposed with TM and normal saline (*n* = 3). LCAs of transplanted B6 heart within TM-treated recipients 2 weeks after grafting remained more patent with minimal obstruction, compared to those of untreated recipients 2 weeks after grafting (Fig. 4a and c). The transplanted B6 heart 4 weeks after grafting showed signs of fibrosis, and all the coronary arteries were stenotic. In the TM treatment group after 4 weeks, similar to the group after 2 weeks, the lumen area was preserved, and SI remained below 50% (Fig. 4b and d).

### Histologic features of cardiac allografts in mice exposed with TM

**Fig. 5:**
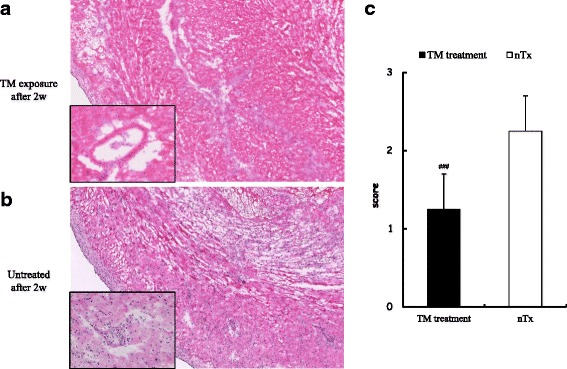
Histologic features in in cardiac allograft exposed with Thrombomodulin (TM) or normal saline. **a**, **b** Results of hematoxylin-eosin staining of myocardium and coronary arteries in cardiac allografts obtained 2 weeks after transplantation from TM-exposed (**a**) or untreated recipients (**b**) (original magnification of bigger panels and smaller panels, × 20 and × 40, respectively). **c** The extent of mononuclear cell infiltration. Each section was assessed by grading with a semi-quantitative scale for the amount of mononuclear cell infiltration (0, no infiltration; 1, faint and limited infiltration; 2, moderate infiltration; 3, severe infiltration). nTx, untreated . ###*p* < 0.001 compared with untreated group

Histologic examinations of cardiac allografts obtained 2 weeks after grafting showed preserved myocardial and vessel structure in TM-exposed recipients, whereas the fracture image of myocardial tissue of allografts from untreated recipients showed the progression of acute rejection (Fig. 5a and b). Moreover, there was a significant difference in the graft score grade on a semi-quantitative scale between the untreated and TM-treated allografts (Fig. 5c).

**Fig. 6:**
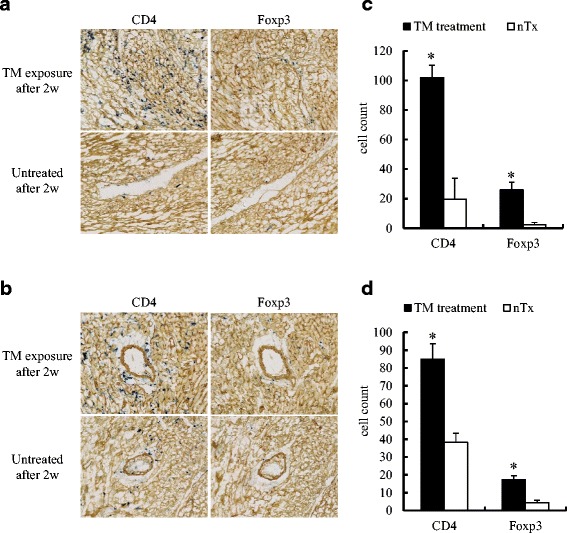
Immunohistochemical features in cardiac allograft exposed with Thrombomodulin (TM) or normal saline. **a**, **b** Results of double immunostaining of myocardium (**a**) and coronary arteries (**b**) in cardiac allografts obtained 2 weeks after transplantation from TM-exposed or untreated recipients (original magnification of all panels, × 40). **c**, **d** The right-hand graphs show the number of infiltrating CD4^+^ and Foxp3^+^ cells in an area of 400 μm × 400 μm of myocardium (**c**) and coronary arteries (**d**) in cardiac allograft from each group. nTx, untreated. *p < 0.001 for difference between two groups

**Fig. 7:**
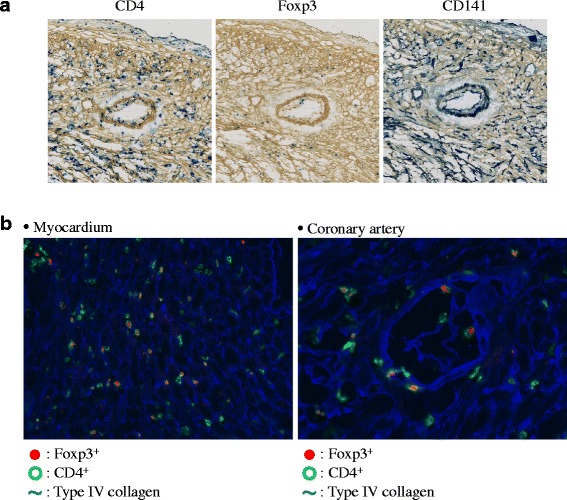
Immunohistochemical features and triple fluorescent staining studies of cardiac allograft exposed with Thrombomodulin (TM). **a** Results of double immunostaining of cardiac allografts obtained 4 weeks after transplantation from TM-exposed recipients (original magnification of all panels, × 40). **b** Triple fluorescent staining studies of cardiac allografts from TM-exposed CBA recipients were assessed 4 weeks after grafting (magnifications of the left- and right-hand panel, × 80 and × 100, respectively). Foxp3, CD4 and type IV collagen are shown as red, green and blue, respectively

IHC examinations of cardiac allografts obtained 2 weeks after grafting showed considerable induction and infiltration of regulatory T cell-like CD4^+^Foxp3^+^ cells in allografts of TM-exposed recipients, whereas allografts from untreated recipients showed more aggressive inflammation, minimal infiltration of CD4^+^Foxp3^+^ cells, and signs indicating progression of acute rejection (Fig. 6a and c). Although TM-treated CBA recipients 2 weeks after grafting showed some progression of intimal thickening of coronary arteries in their allografts, the count of infiltration of CD4^+^Foxp3^+^ cells around coronary arteries was significantly more than that of untreated mice. Conversely, some vessels in allografts from untreated recipients were stenosed by 50 to 80% or completely obstructed, had less CD4^+^Foxp3^+^ cells, and strong filtration of CD4^+^Foxp3^−^ cells (Fig. 6b and d). Histologic features after 4 weeks demonstrated clear CD4^+^Foxp3^+^ and CD141^+^ expression on the inner surface of coronary arteries within allografts from TM-exposed recipients relative to those of untreated recipients (Fig. 7a).

### Prominent accumulation of CD4^+^Foxp3^+^ cells in cardiac allografts from TM-exposed mice

Fluorescent staining studies done 4 weeks after grafting clearly demonstrated that cardiac allografts from TM-exposed CBA recipients had aggregation of CD4^+^Foxp3^+^ cell in the myocardium of allograft (Fig. 7a). Notable, infiltration of CD4^+^Foxp3^+^ cells to the intima of the coronary arteries in transplanted B6 hearts was observed (Fig. 7b).

## Discussion

The present study, demonstrated for the first time that TM administration during cardiac allograft transplantation can have graft-protective effects and induce CD4^+^Foxp3^+^ T cells within the allograft to prevent acute rejection in a murine fully MHC-mismatched model. There are several possible mechanisms by which TM administration could induce prolongation of murine cardiac allografts. Firstly, one mechanism may be through the suppression of intimal hyperplasia and arteriosclerosis within the coronary arteries of the allograft. This is supported by previous studies that have elucidated its activity within transplantation-associated micro-angiopathy [5] and engraftment syndrome after hematopoietic stem cell transplantation [10]. Moreover, another report has demonstrated that TM overexpression limits neointimal formation in common femoral arteries of the rabbit model [11]. In our IHC study, the TM-treated group demonstrated preserved myocardial structure at 2 weeks post-transplantation and showed a significant statistical difference compared to the control group that had all rejected their grafts by 2 weeks (Fig. 5). Additionally, the vascular structure within the coronary artery in the TM treatment group was maintained and infiltration of inflammatory cells such as monocytes into the coronary arteries was suppressed (Fig. 5). In concordance with our IHC results, both the LCA and RCA in the control group 2 weeks after grafting were narrowed by an average of 60 to 80%, whereas the stenosis rate of the TM treatment group was limited to about 30% (Fig. 4). This disparity was even more distinct after 4 weeks. The allografts of the untreated group 4 weeks after grafting showed progression of fibrosis and almost all the coronary arteries were occluded, whereas TM treatment held the coronary stenosis rate at 40 to 50%, suggesting that the graft protective effect could continue up to 3 weeks after administration of TM. However, there were no findings to suggest that the effect of TM remained beyond 3 weeks from the end of TM administration, so it was conceivable that there would be other mechanisms in play to explain the vascular protective effects after 4 weeks post-engraftment.

Our previous studies have demonstrated that substantial accumulation of inducible CD4^+^Foxp3^+^ regulatory T cells was observed around the coronary arteries of prolonged grafts [12–14]. Thus, based on the previous findings, it was reasoned that a second mechanism by which TM can protect the graft is through the generation of regulatory CD4^+^Foxp3^+^ T cell. From the IHC staining, it was observed that CD4^+^Foxp3^+^ cells accumulated in the myocardium and around the coronary arteries of allografts in the TM-treated recipients at 2 weeks after grafting (Fig. 6). After 4 weeks, while assessment of pathological staining became difficult in the untreated group due to severe fibrosis, CD4^+^Foxp3^+^ cells and CD141 expression in the myocardium and around the coronary arteries of allografts within TM-treated recipients were maintained while it was not in the untreated group. TM’s effect on CD4^+^Foxp3^+^ cells has also been documented in previous reports. rTM administration to murine acute respiratory distress syndrome (ARDS) model has been shown to induce prolongation of survival time and ameliorate development of ARDS through increasing regulatory T cell in the lung [15]. Moreover, in another study, it was shown to alleviate GVHD via increasing splenic regulatory T cell [16]. Based on our previous data, it can be reasoned that inducible CD4^+^Foxp3^+^ Treg do not disappear in a short period of time [17, 18]. From the findings in this study that demonstrate that the general structure of the allograft was maintained even three weeks after termination of TM administration, it can be concluded that TM may generate CD4^+^Foxp3^+^ regulatory T cells that infiltrate transplanted cardiac allografts and that greater aggregation of these cells around coronary arteries may lead to the prolongation of allograft survival.

There are some limitations to this study. First, 50% of TM-treated recipients did not show the prolongation of allograft for more than 100 days after grafting. We determined the term of 7 days of TM administration according to previous studies on TM treatment for DIC that have only administered TM up to 7 days from the onset of DIC and the fact that 90% of our transplanted recipients rejected allografts acutely within 8 days. Future work which extends the administration period or optimizes TM dosage may yield enlightening results. Second, the correlation between blood concentration of TM administrated and graft survival after TM administration is unknown. Considering the minute difference of MST, it might be necessary to evaluate the TM concentration after administration in time series. Finally, the present study was conducted using a small number of mice with a limited follow-up period. If the efficiency of TM pointed out by this study is observed in the long-term, a longer observation period and detailed analysis of these recipients is required.

## Conclusion

In summary, TM prolonged survival of fully MHC-mismatched cardiac allograft through the suppression of intimal hyperplasia and inducing the accumulation of regulatory CD4^+^Foxp3^+^ cell within the coronary arteries. Further research which optimizes the TM regimen after allograft transplantation may lead to even greater outcomes.

## Abbreviations

ALP: Alkaline phosphatase
C57BL/6: B6
DIC: Disseminated intravascular coagulation
HE: Hematoxylin-eosin
IHC: Immunohistochemical
LCA: Left coronary artery
mAb: Monoclonal antibody
MHC: Major histocompatibility complex
MST: Median survival time
PC: Protein C
RCA: Right coronary artery
rTM: Recombinant human soluble thrombomodulin
SI: Stenosis index
TM: Thrombomodulin
